# A Narrative Review of the Use of Artificial Intelligence in Breast, Lung, and Prostate Cancer

**DOI:** 10.3390/life13102011

**Published:** 2023-10-04

**Authors:** Kishan Patel, Sherry Huang, Arnav Rashid, Bino Varghese, Ali Gholamrezanezhad

**Affiliations:** 1 Department of Radiology, Keck School of Medicine, University of Southern California, Los Angeles, CA 90033, USAali.gholamrezanezhad@med.usc.edu (A.G.); 2 Department of Urology, University of Pittsburgh Medical Center, Pittsburgh, PA 15213, USA; 3 Department of Biological Sciences, Dana and David Dornsife College of Letters, Arts and Sciences, University of Southern California, Los Angeles, CA 90089, USA

**Keywords:** radiology, artificial intelligence, machine learning, breast cancer, prostate cancer, lung cancer, cancer screening

## Abstract

Artificial intelligence (AI) has been an important topic within radiology. Currently, AI is used clinically to assist with the detection of lesions through detection systems. However, a number of recent studies have demonstrated the increased value of neural networks in radiology. With an increasing number of screening requirements for cancers, this review aims to study the accuracy of the numerous AI models used in the detection and diagnosis of breast, lung, and prostate cancers. This study summarizes pertinent findings from reviewed articles and provides analysis on the relevancy to clinical radiology. This study found that whereas AI is showing continual improvement in radiology, AI alone does not surpass the effectiveness of a radiologist. Additionally, it was found that there are multiple variations on how AI should be integrated with a radiologist’s workflow.

## 1. Introduction

Artificial intelligence (AI) refers to the ability for a machine to simulate human intelligence to perform tasks involving decision making and problem solving. As in other industries, AI technologies have found widespread applications in a variety of healthcare tasks including, but not limited to, analyzing unstructured clinical notes, developing clinical decision support systems, innovating surgical robotics, and establishing patient engagement and adherence. Within radiology, AI applications have been limited to specific image recognition tasks associated with patient management [1]. Table 1 provides a brief definition of AI and other AI-related terms referenced.

Two of the more common AI technologies in healthcare are machine learning (ML) and deep learning (DL). Machine learning refers to the ability for a machine to develop algorithms that make predictions on data based on trends and patterns from previous data [2]. Deep learning is a subset of machine learning that involves layered learning and model learning via neural networks and allows for predictions in unstructured environments [3]. One of the more common architectures of the AI models seen in imaging analysis is convolutional neural networks (CNN) or deep convolutional neural networks (DCNN). CNNs allow for the generalization of features and the extraction of such features in a superior way to prior deep learning models [4]. This allows CNNs to be efficient and effective in finding patterns and building models from such patterns.

The use of AI within radiological imaging is growing rapidly, especially in the field of radiomics [5]. Radiomics refers to the extraction of features from images such as shape, size, and texture [6]. These features can be utilized in an algorithm to provide diagnostic support for a number of conditions. One of the conditions where radiomics is heavily utilized is cancer imaging [7,8]. The use of radiomics can be varied from its use in screening to its utilization in predicting tumor burden and therapy guidance. The three most common cancer diagnoses in the United States are lung, breast, and prostate cancer [9]. The diagnosis and management of such cancers are multifaceted with the involvement of radiologists, oncologists, and surgeons. Radiomics and AI may assist in the diagnosis and management of each of these cancers. The use of radiomics and AI in cancers has been seen for nodule classification, tumor description, metastatic potential, and treatment response [10].

Despite the wide use potential of radiomics and the numerous types of AI software developed, few software packages have been clinically validated and even fewer have been implemented in a radiologist’s workflow. The clinical validity of AI, however, has seen growth in lung cancer screening programs [11]. Radiomics in prostate cancer imaging has been used for the detection of tumor lesions, lymph node metastasis, and patient risk stratification [12]. Meanwhile, AI’s use in breast cancer has been seen most predominantly in breast cancer mammography screening programs. However, the use of AI has also been seen in magnetic resonance (MR) imaging and ultrasound imaging [13,14].

The goal of this article is to provide a review of the available technologies as well as the present studies testing the validity and accuracy of AI software in breast, prostate, and lung cancer screening and diagnosis.

## 2. Materials and Methods

A literature review was conducted between the dates of 1 April 2023 and 30 July 2023 by three independent reviewers on PubMed and Google Scholar. The following individual terms were entered as search criteria: “artificial intelligence”, “AI”, “machine learning”, “deep learning”, “radiomics”, “prostate”, “breast”, “lung”, “cancer”, “screening”, “mammogram”, “MRI”, “CT”, “ultrasound”, “diagnosis system”, “decision system”, “CNN”, and “deep learning”. The same terms were searched in both databases.

From the searched criteria, only articles published in 2018 or later were included for initial review. A 5-year period (2018–2023) was utilized, as the continuous growth rate of AI research in radiology is approximately 50% for this period. This indicates a significant growth in AI research during this time frame [15]. The abstracts and manuscripts were analyzed for relevance to goals of the current review article. Articles that did not include the measures of sensitivity, specificity, or accuracy of an AI model were not included for review. Articles were additionally only included if statistical measures were performed, and size of the study was appropriate. No restrictions were made regarding the location of where the study was performed.

No statistical analysis was performed by the authors of this article. All included statistical measures were taken from the respective cited manuscripts. Multiple measures of accuracy were included within the manuscript and described within the relevant section. Because all data and studies were sourced from differing imaging databases, the lack of standardization and inability to therefore perform statistical analysis represents a limitation of this review.

## 3. Breast Cancer Screening

The use of AI to assist in lesion detection has been seen in breast lesion imaging. AI’s use has been seen in mammography, magnetic resonance imaging (MRI), and the ultrasonography of breasts. Mammography is the most utilized imaging modality for breast imaging and plays a pivotal role in the early detection and diagnosis of breast cancer [16]. MRI has been used as a screening tool in patients with dense tissue mass or those with a high risk of developing breast cancer [17]. Ultrasonography may also be used for the screening of high-risk patients who are unable to tolerate MRI [18]. The greatest number of ML and DL models have been developed for mammography. Although some models have also been developed for MRI and ultrasound, the clinical utility of these tools has yet to be fully determined. AI tools can be used to assist in either the detection or diagnosis of breast lesions. Detection systems do not make a diagnosis but rather mark areas of the image that seem suspicious [19]. The goal of these systems is to point the radiologist to areas of high concern (such as calcifications, masses, or parenchymal distortions) and notify them that such areas may need extra attention in reading. Diagnosis systems take detection systems one step further and classify suspicious findings as either benign or malignant lesions [20]. Detection systems typically work by processing and enhancing the given image, followed by selecting and extracting features through pattern recognition. These steps involve the use of neural networks or ML or DL algorithms developed from training models [21]. Decision systems follow the above processes but use additional algorithms to classify the lesions.

A study comparing several different AI algorithms found that from 12 models, 10 models were over 90% accurate in diagnosing breast lesions as either benign or malignant. The range of accuracies ranged from 85.5% to 97.8% [22]. A large study of 122,969 breast mammograms from Norway utilized an AI software developed by ScreenPoint Medical that graded both interval and screen-detected mammograms on a scale of 1–10, where 10 indicates a lesion is most likely to be malignant. The study found that 86.8% of the screen-detected cancers and 44.9% of the interval cancers were given a score of 10. Additionally, 2.3% of screen-detected cancers and 19.1% of interval cancers were given a score of 5 or lower [23]. In another study from Turkey, a retrospective analysis of 211 mammograms using the Lunit INSIGHT software found that 83.8% of screen-detected cancers were given a risk score above 34.5%, whereas 44.4% of interval cancers were given a risk score above 34.5% [24]. In this study, the cancer detection rate was 67.3% for the radiologist, 72.7% for the AI software, and 83.6% when the software and radiologist both interpreted the imaging, which is evidence supporting the added value of AI in clinical interpretation. A validation study of the Saige-Q software developed by Deep Health on Australian patients found that the software positively marked 76.8% of screen-detected cancers and 36.6% of interval cancers [25]. Further analysis demonstrated that the detection rate was equivalent for screen-detected ductal carcinoma in situ (DCIS) and invasive carcinoma. A study from the German national breast cancer screening program performed an analysis of 4463 screen-detected cancers and 100,005 normal studies using AI. The sensitivity and specificity of the AI system alone were 2.6% and 2.0% lower than that of a reading radiologist, respectively. However, the use of both an AI system and a reading radiologist increased the sensitivity and specificity by 2.6% and 1.0%, respectively, when compared with a sole reading radiologist [26]. Other studies have similarly shown that AI is less sensitive and specific compared with a radiologist. A review of a number of AI mammography software packages showed that 94% of the tested pieces of software were less accurate than a single reading radiologist and 100% were less accurate than two reading radiologists [27].

Studies have also shown the accuracy of AI software in breast MRI and ultrasound. In a study using QuantX’s computer-aided diagnosis software, the average area under the curve (AUC) was compared for images with and without AI assistance. It was found that the AUC was higher when AI software and a reading radiologist were used together (0.76) versus when a radiologist alone was used (0.71) [28]. A review of AI in breast MRI looked at a number of different algorithms and found that the median AUC for prognostic imaging was 0.80 and median AUC for neoadjuvant therapy response was 0.85 [29]. In a review of AI in ultrasound, it was found that the AUC for all studies utilizing an AI model was above 0.8. Additionally, in some studies it was found that the use of AI in ultrasound prevented the need for unnecessary biopsies in patients with suspected BIRADS 4A lesions [30].

**Figure 1:**
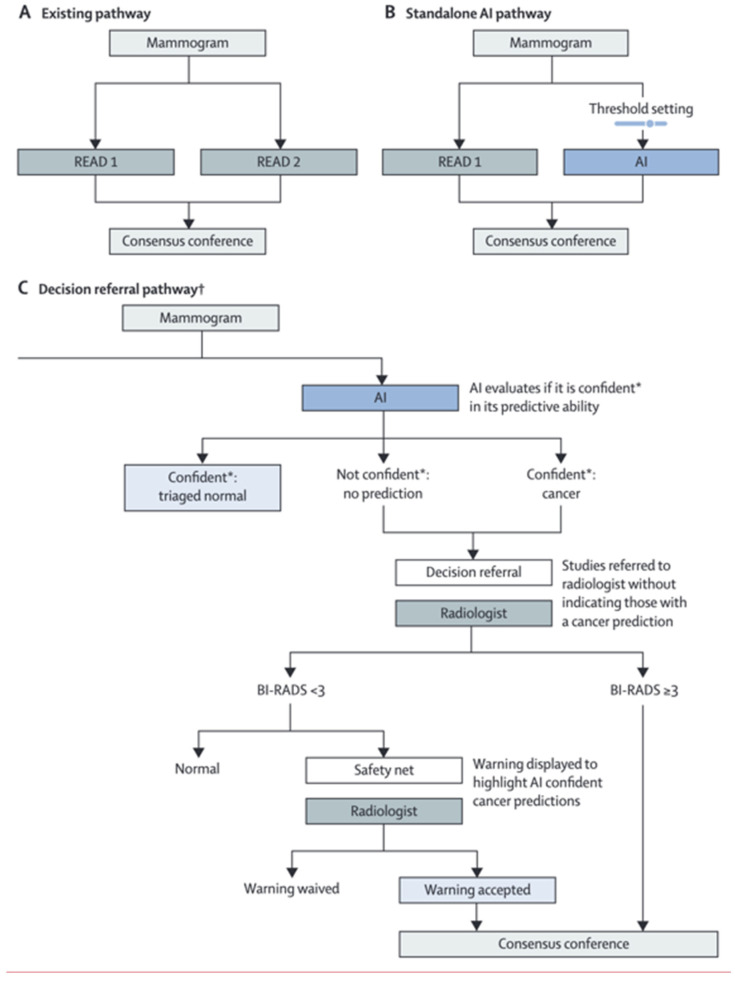
Comparison between the decision and referral and standalone AI pathway in double-reader screening settings. † Decision-referral pathway utilizing AI and one reading radiologist. * Confidence of the AI model in determining if a lesion is malignant or not. From Leibig C et al. [26].

The effectiveness of AI in breast screening has led to non-converging results. In some studies, AI is shown to provide better detection rates than a reading radiologist, whereas in others it is shown to be less effective. This may stem from the poor consensus in implementing AI approaches. From the studies of AI utilization in mammography, US, and MRI, it appears that although AI may act as a supplement to a radiologist, the models are not accurate enough to replace a radiologist. Utilizing AI as an additional screening tool before a radiologist reads the image or as a checking tool after a radiologist interprets the image may allow for AI to improve the overall reading accuracy. Moreover, how AI should be utilized in a screening program is not well demonstrated. Currently, in some screening programs, a mammogram is read by two radiologists before a decision is made. However, with AI it is possible to allow AI to act as the second reader, meaning that only one radiologist would be needed. Figure 1 demonstrates the possible pathways for AI in breast imaging [26]. Although studies have shown that using one radiologist with AI generally produces more sensitive and specific reads compared with a radiologist alone, few studies demonstrate the outcomes when comparing AI with two reading radiologists. Furthermore, the use of AI on interval cancers is consistently less accurate than with screen-detected cancers, which may prevent accurate reads if AI were to replace a second reading radiologist.

In MRI and US, AI has also been shown to have positive outcomes in improvement of the AUC [29,30]. However, similar to mammography studies, the imaging criteria for cutoffs vary. Additionally, studies are more limited for both MRI and ultrasound and datasets are generally smaller, indicating the results may not be as reproducible.

## 4. Lung Cancer Screening

Lung cancer is the leading cause of cancer deaths around the world annually [31]. Although there are multiple standards for the detection of lung nodules on CT imaging, such as Lung-RADS or Fleischer Society criteria, lung cancer may often be missed on imaging, especially on plain films. One study found that nearly 90% of missed lung cancers occur on chest X-ray [32]. Furthermore, chest X-rays are one of the most frequently requested radiological imaging studies worldwide. Some studies have shown that increased rates of chest X-ray imaging can lead to earlier lung cancer detection and improved patient outcomes [33]. Recently, the use of AI in lung cancer imaging has come into the discussion. Numerous ML and neural network models have been developed, some of which have shown high sensitivity and accuracy for the detection of lung nodules [31].

In one multi-center international study utilizing the Samsung Auto Lung Nodule Detection DCNN software, it was found that utilizing both reading radiologists and the AI software led to a 5% increase in sensitivity and a significant decrease in the number of false positives for the detection of lung nodules on chest radiographs [34]. In this study, radiologists were asked to review a set of images and mark areas of suspicious nodules. The images were later read again by the same radiologists after the algorithm had marked areas of interest. Radiologists were then tasked with either accepting or rejecting the algorithm’s suggestions. In another study of chest plain films from the United Kingdom, it was found that use of a DCNN model alongside radiologists led to a 60% reduction in the number of missed lung cancers [31]. When the model was used alone, the sensitivity was 80% and the specificity was 93%, lower than each of the three reading radiologists in the study. The number of false positives from the DCNN software was also significantly greater than the radiologists.

In a study using another commercially available DCNN model, the effect of AI as a second reader differed between radiology residents and radiology attendings. When utilizing AI as a second reader, sensitivity improved for the radiology residents, whereas specificity improved for attending radiologists [35]. Thus, the AI software helped more novice residents find difficult to find lesions, such as those overlapping with mediastinal structures or vasculature. For attendings, the software helped exclude initially uncertain lesions more confidently. The benefit of an algorithmic model appears to differ based on skill level; however, it is apparent that there is a benefit to radiologists when the AI model functions as a second reader.

When a suspicious nodule is found on chest X-ray, it is likely to be followed up with either a low-dose CT scan (LDCT) or regular CT scan [36]. Additionally, solitary pulmonary nodules may be found incidentally on CT imaging conducted on patients. These nodules may require regular follow-up based on the Fleischner criteria [37]. AI models have also been developed for the detection and classification of nodules found in CT imaging. There have been numerous neural network models utilized and researched for the detection of lung nodules in CT, including regional CNN (RCNN), multi-resolution CNN (MRCNN), and hierarchical saliency CNN (HSCNN) among others [38]. Newer models are continually being developed to improve the accuracy of detection. For instance, in one 2020 study analyzing two datasets, each with over 800 chest CT images, a novel deep convolutional neural network (DCNN) was compared with older RCNN, MRCNN, and HSCNN models. Each image was read by four radiologists and the nodules were annotated. The neural network models were tested against each other and the accuracy was compared with the radiologists. It was found that the accuracy, sensitivity, and specificity for nodule detection were significantly improved for the novel DCNN model [38]. In another study of a commercially available DL-CAD software, nodule detection was compared between two radiologists (double-reading group) and the DL-CAD software. The software was seen to be statistically more significant for nodule detection of all sizes when compared with the radiologist pair [39]. However, the rate of false positives was also significantly higher in the DL-CAD group. This study indicates that CAD software may be proficient as a screening tool for lung nodules. Although these studies did not contain diagnosis systems for lung cancer, they indicate the continual improvement in neural network models in feature detection.

In the United States, the USPSTF recommends yearly LDCT for lung cancer screening in certain individuals with extensive smoking history (i.e., adults 50–80 years old with a 20 pack-year smoking history, current smoker, or those who have quit within the past 15 years) [40]. Additionally, many countries have begun discussing lung cancer screening with LDCT, which may result in a significant increase in LDCT volume. With the high volumes of LDCT anticipated, an AI model may be beneficial to use as a screening tool for LDCT images. In a study utilizing an RCNN model to investigate over 6700 LDCT screening cases from the National Lung Cancer Screening Trial dataset, it was found that the model led to reductions in false positive and false negative rates [41]. The study was conducted for individuals both with prior LDCT screening imaging and those without. The study found that in cases where there was no prior LDCT imaging, the AI model had an 11% reduction in false positives and a 5% reduction in false negatives. When there was prior LDCT imaging, there was no significant difference in false positive and negative rates between the radiologist and model [41]. This study indicates that an AI model may be sufficient as an initial screening filter prior to the image being read by a radiologist.

Some software packages have also aimed to utilize radiomic data to predict the genotypic variation of lung nodules. For instance, the epidermal growth factor receptor (EGFR) mutation may be seen in certain non-small cell lung cancers (NSCLCs) [42]. Optimal pharmaceutical treatment may vary based on specific EGFR mutation types; thus, the classification of the specific EGFR genotype may be of increased importance. One study utilized a DL model to study both nodule features and whole-lung features. The model achieved over 65% accuracy in correctly detecting the EGFR genotype among all tested data sets [43]. By utilizing radiomic features both within and outside a pulmonary nodule, AI models may be able to better predict tumor mutations and genotypes prior to lesion biopsy.

**Figure 2:**
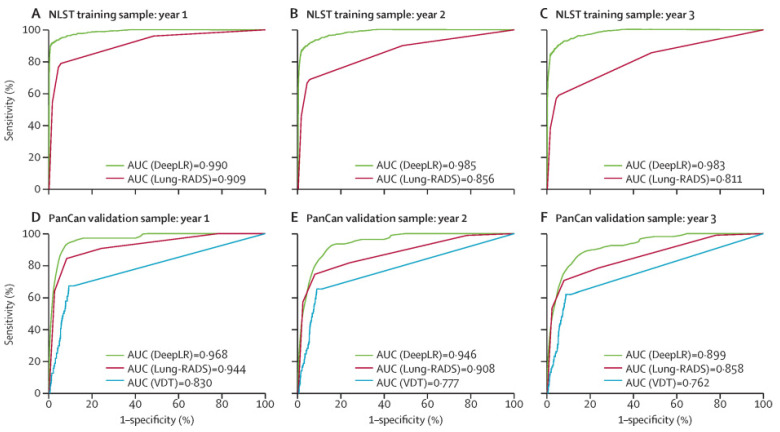
Upper panels: comparison of AUC values for DeepLR and Lung-RADS in the NLST training cohort (n = 25 097) at 1 year (**A**), 2 years (**B**), and 3 years (**C**) after the S2 scan. Lower panels: comparison of AUC values for DeepLR, Lung-RADS and VDT in the the PanCan validation cohort (n = 2294) at 1 year (**D**), 2 years (**E**), and 3 years (**F**) after the S2 scan. NLST = National Lung Screening Trial. Lung-RADS = Lung CT Screening Reporting and Data System. VDT = volume doubling time. AUC = area under the receiver operating characteristic curve. PanCan = Pan-Canadian Early Detection of Lung Cancer Study. S2 = last CT screening without cancer diagnosis. From Huang P et al., [44].

Beyond nodule feature detection, models have been developed to improve upon current imaging guidelines, such as those set forth by Lung-RADS. The DeepLR model developed at Johns Hopkins was shown to more accurately predict the risk of malignancy development at one, two, and three years’ post-LDCT imaging than the imaging criteria set forth by Lung-RADS [44]. Similar to the Lung-RADS criteria, the DeepLR model considered nodule features; however, extranodular features such as the presence of cardiac disease or emphysema were also considered. Additionally, the DeepLR model further considered other nodular features such as a change in attenuation, location, or nodule margins when building the model. When comparing the specificity of diagnosis of lung cancer using DeepLR vs. Lung-RADS, DeepLR was seen superior at the one-, two-, and three-year timepoints (Figure 2).

For patients found to have a suspicious lesion over 8 mm in size on LDCT screening, there does not exist a definite clinical decision pathway for follow-up. Some recommendations included a 3-month follow-up LDCT to measure for volumetric and size change, tissue sampling, or ^18^fluorodeoxyglucose (FDG) positron emission tomography–computed tomography (PET/CT) [37]. In a study of Swedish PET/CT images, a dual CNN system was used to characterize and segment lung nodules found on PET/CT. The CNN utilized in the CT imaging was used to segment organs, which would demonstrate high FDG uptake and hypermetabolism on PET/CT. The other CNN was used with both the CT and PET imaging to classify lung lesions from non-lesions. The study found that the combined CNN models resulted in 90% sensitivity in detecting abnormal lung lesions [45]. The model was noted to have lower segmentation accuracy when the lesions were located more medially or when lesions contained necrotic components [45]. Whereas intra- and inter-observer reliability is generally higher for the PET/CT imaging of lung nodules compared with CT, a CNN AI model may assist in a screening tool for nuclear medicine specialists.

The use of AI has been well studied in lung cancer. Research tools have been developed for X-ray, CT, and PET imaging. Furthermore, with the growth of lung cancer diagnosis and further regulations on screening, a highly sensitive CNN model may be an effective tool in serving as a first-line screening agent for lung cancer imaging.

## 5. Prostate Cancer Screening

**Figure 3:**
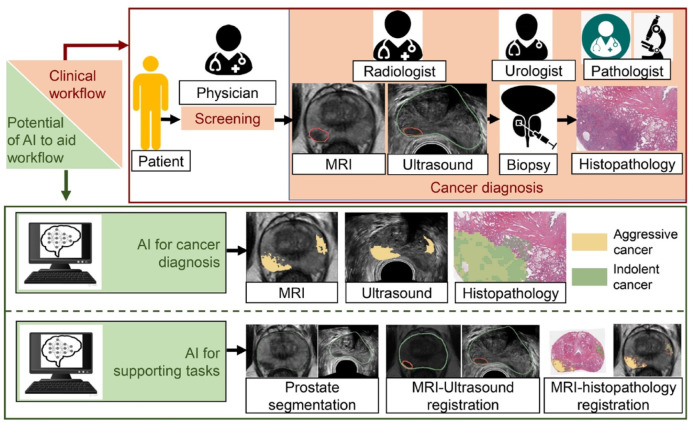
Potential of AI to assist prostate cancer diagnosis via imaging. AI models can help in detecting and characterizing cancer aggressiveness on non-invasive radiology images (MRI and ultrasound), as well as on histopathology images acquired through prostate biopsy. Aggressive cancer is shown in yellow and indolent cancer in green in the “AI for cancer diagnosis” panel. AI models can also help in supporting tasks for cancer detection, namely prostate gland segmentation, MRI–ultrasound registration, and MRI–histopathology registration. From Bhattacharya et al. [48].

In the United States, prostate cancer is the most common non-cutaneous malignancy and the second leading cause of cancer death in men [9,46]. It is the sixth most common cancer worldwide and is often diagnosed by a prostate biopsy and graded according to the Gleason scale [46,47]. Other forms of screening and diagnostics include the prostate-specific antigen (PSA) blood test, MRI imaging of the prostate, and newer tests including urine biomarkers and genetic testing. After all the information is collected, the prostate cancer is staged from low-risk to high-risk, with wide variation in the intermediate-risk category [46,48]. It is important to limit the variation of pathological scoring and proper radiological detection of prostate cancer tumors, as this information can inform methods of treatment—active surveillance, radiation, surgical excision, or a combination of multiple therapies [46,49]. Artificial intelligence has shown promising results in improving the detection and grading of prostate cancer, which would help ensure that patients are receiving the best treatment for their stage of cancer. Figure 3 demonstrates the multiple uses of AI in prostate cancer imaging [48].

MRI is a tremendously important tool in the diagnosis of prostate cancer, both for the initial detection and for MRI–ultrasound fusion biopsies of lesions. In terms of the detection and localization of the cancer, multiparametric MRI (mpMRI) is the most sensitive non-invasive method for identifying prostate cancer; however, there still exists variation in the subtle interpretations of visual cues [48]. Sonn et al. evaluated the mpMRI scans of 409 patients and found high variability of inter-rater and intra-rater radiologic assessment using PI-RADS (Prostate Imaging Reporting and Data System) [50]. AI can be utilized here to improve detection of prostate cancer that is not easily visible to the naked eye. One study by Lay et al., using a computer-aided diagnosis (CAD) model, achieved an AUC score of 0.93 in distinguishing low-grade from high-grade prostate cancer [51]. Other studies using CAD have achieved AUC scores from 0.80 to 0.96, showcasing improvements in inter-rater reliability and accuracy, though some of these studies may lack generalizability [52]. Hiremath et al. performed a retrospective multicenter study and constructed an integrated nomogram using a DL model, PI-RADS, and clinical attributes to risk stratify prostate cancer according to mpMRI. With nearly 600 patients total, the nomogram achieved an AUC of 0.81 when detecting prostate cancer and developed significantly different Kaplan–Meier curves when measuring biochemical recurrence in patients, performing better than a solely deep-learning-based predictor or the PI-RADS model [53].

Li et al. conducted a retrospective analysis of 203 patients using a radiomics model trained on mpMRI and PI-RADS to distinguish between patients with and without prostate cancer. The radiomics model combined with PI-RADS was significantly better than using PI-RADS alone for diagnosing prostate cancer (AUC of 0.93) [54]. Another recent study of AI and mpMRI detected the extracapsular extension (ECE) of prostate cancer with an AUC range of 0.728–0.857, which was higher than assessments by two experts, which had an AUC range of 0.632–0.857 [55]. Improving detection of ECE can help to inform surgical planning or guide certain radiotherapies. CAD, radiomics, and DL algorithms have great potential to improve the detection and diagnosis of prostate cancer while limiting errors and decreasing variability.

The interplay between radiological screening, urological intervention (i.e., biopsies, prostatectomy), and histopathology is the key to the accurate diagnosis and staging of prostate cancer. There have been some indications that AI algorithms can decrease the variability of Gleason scoring across pathologists, which can improve radiology–pathology correlation of detecting prostate cancer. One difficulty in applying machine learning algorithms to Gleason grading is due to the subjectivity of the system, especially for intermediate-to-high risk prostate cancer, which potentiates issues when generating a training dataset for the model to properly classify the Gleason grade [46]. Although automated detection of tissue components has been proven to be helpful in determining prognoses for patients with breast cancer, there is still work to be performed for prostate cancer, in part due to the heterogeneity of the tissue samples and labeling.

Strom et al. trained an AI algorithm to distinguish benign and malignant biopsies of nearly 1000 patients and achieved an AUC of 0.997 [56]. Another study evaluated the use of an AI prostate biopsy cancer detection system for whole-slide imaging compared with an expert alone, which resulted in a significant increase in sensitivity from 74% (expert alone) to 90% [57]. It is important that histopathological readings of prostate tissue are as accurate as possible, as these are the foundation for training data and the key to improving AI models, which can then be extended to improving the non-invasive radiological screening and diagnostic methods. Furthermore, AI applications correlating tissue-level components of the prostate with MRI signals have shown promising results for guiding prognosis. According to one study by McGarry et al., combining mpMRI and histopathology indicated regions of epithelium and lumen density differences that correlated better with post-prostatectomy-confirmed high-grade prostate cancer [58]. Another study utilizing hybrid multidimensional MRI and pathology after prostatectomy identified AUCs of 0.991, 0.800, and 0.789 for differentiation of epithelium, lumen, and stroma, respectively, when comparing malignant tissue with non-cancerous tissue [59]. There is great value in utilizing AI to understand and further investigate the radiologic–pathologic correlation of grading prostate cancer, as combining information from both disciplines can give a more complete picture.

There are several challenges that exist prohibiting the widespread use of AI for prostate cancer. First, there is a lack of generalizability due to overfitting of the AI models. This is largely due to the lack of widespread, public data when training the models, resulting in smaller sample sizes and models that are overly specific to their training data set [60]. There are also imbalances in the samples. For example, many studies utilize lesions that are located in the peripheral zone of the prostate rather than other zones (transition, central, etc.). This is an important consideration because it will contribute to the lack of variability within the training dataset and therefore lead to more overfitting. There is an effort to address this, as Mehralivand et al. performed a multicenter study that showed minimal increase in sensitivity overall when using AI to compare benign and malignant lesions on mpMRI but demonstrated a statistically significant increase in the sensitivity for identifying cancerous lesions in the transition zone of the prostate [61].

Another challenge with adopting the use of AI is lack of standardization and reproducibility of the research and protocols [60]. There is an increased effort to conduct more multi-center studies to attempt to overcome the lack of standardization and ability to generalize the results. Recent studies have indicated strong results utilizing CAD with mpMRI across multiple institutions, achieving high AUC scores (0.81–0.96) [62]. Despite these efforts, however, there is still a lack of reproducibility in the research, as there is no standardized method to report specific information regarding protocols such as guidelines on how to use the datasets [60]. Ultimately there are a variety of factors that contribute to obstacles when employing AI in prostate cancer—lack of high-quality data, lack of available data, operating costs, lack of validation, etc. [46]. More high-quality research showcasing proven methodologies should be conducted to fully unlock the potential that AI can bring to assist clinicians in diagnosing and detecting prostate cancer.

Despite this host of challenges, there is great potential for adapting AI into the diagnostic process for prostate cancer. There are many new avenues of research being undertaken. The combination of newer techniques and imaging modalities with AI can provide novel methods to improve the accuracy and efficiency for detecting prostate cancer. AI combined with tools such as the PSMA PET (Prostate Specific Membrane Antigen Positron Emission Tomography) for focal localization of prostate cancer and metastases, TRUS (transrectal ultrasound scan) fusion biopsies with MRI for superior prostate mapping and radiotherapy, and radiogenomics to identify new genetic biomarkers are only a few hot areas for exploration [63,64].

## 6. Summary

Radiological imaging plays an important role in the screening and diagnosis of breast, lung, and prostate cancers. With an aging population and guidelines recommending screening be started at an earlier age, the need for rapid and accurate screening grows more critical. During the past few years, there have been numerous ML and DL models researched that address this issue. Many of these models use DCNN algorithms, which allow for superior image recognition. This study reviewed the accuracy and effectiveness of a number of AI models created within the past few years on numerous modalities. Additionally, this study reviewed manuscripts utilizing numerous different DCNN algorithms. The findings of this review indicate that although AI does perform well on its own for imaging analysis, in almost all cases the combination of using a trained radiologist and an AI model provides superior benefit than either one used individually. Additionally, AI models tend to produce more false positives, which limits their standalone ability in interpreting images. It is apparent that AI has significant benefits for radiologists; however, how to utilize AI within a clinical workflow should be researched further. Whether AI should be used to triage imaging or to catch potential misses by a radiologist should be discussed. Furthermore, the use of AI for training radiologists should be researched, as AI models have shown efficacy in finding lesions that an untrained radiologist may not initially see. Overall, AI has shown tremendous growth in capability over the past few years. Nevertheless, it is still too early to determine the best use of AI in radiology. AI has shown efficacy in improving workflows, diagnosis abilities, and teaching abilities; however, further research must be conducted before a specific utilization use case can be recommended. However, it is evident that with continual improvement AI will play an active role in some form for radiologists.

## Figures and Tables

**Table 1:** Definition of terms relating to artificial intelligence.

Term	Abbreviation	Definition
Artificial Intelligence	AI	An overarching term referring to the ability for a machine to perform intelligent tasks such as decision-making.
Machine Learning	ML	A subset of artificial intelligence referring to the ability for a machine to make predictions based on trends and patterns.
Deep Learning	DL	A subset of machine learning referring to the utilization of neural networks to develop predictions.
Convolutional Neural Network	CNN	A type of algorithm utilized in deep learning that relies on a feed forward mechanism and is utilized in object identification. DCNNs and RCNNs are specific types of CNNs.

## Data Availability

All data may be referenced from the respective cited manuscript.
